# Are microglia minding us? Digging up the unconscious mind-brain relationship from a neuropsychoanalytic approach

**DOI:** 10.3389/fnhum.2013.00013

**Published:** 2013-02-04

**Authors:** Takahiro A. Kato, Shigenobu Kanba

**Affiliations:** ^1^Department of Neuropsychiatry, Graduate School of Medical Sciences, Kyushu UniversityFukuoka, Japan; ^2^Innovation Center for Medical Redox Navigation, Kyushu UniversityFukuoka, Japan

**Keywords:** microglia, psychoanalysis, emotion, stress, unconscious, death instinct, suicide, psychiatry

## Abstract

The unconscious mind-brain relationship remains unresolved. From the perspective of neuroscience, neuronal networks including synapses have been dominantly believed to play crucial roles in human mental activities, while glial contribution to mental activities has long been ignored. Recently, it has been suggested that microglia, glial cells with immunological/inflammatory functions, play important roles in psychiatric disorders. Newly revealed microglial roles, such as constant direct contact with synapses even in the normal brain, have defied the common traditional belief that microglia do not contribute to neuronal networks. Recent human neuroeconomic investigations with healthy volunteers using minocycline, an antibiotic with inhibitory effects on microglial activation, suggest that microglia may unconsciously modulate human social behaviors as “noise.” We herein propose a novel unconscious mind structural system in the brain centering on microglia from a neuropsychoanalytic approach. At least to some extent, microglial activation in the brain may activate unconscious drives as “psychological immune memory/reaction” in the mind, and result in various emotions, traumatic reactions, psychiatric symptoms including suicidal behaviors, and (psychoanalytic) transference during interpersonal relationships. Microglia have the potential to bridge the huge gap between neuroscience, biological psychiatry, psychology and psychoanalysis as a key player to connect the conscious and the unconscious world.

## Introduction

“We have often heard it maintained that sciences should be built up on clear and sharply defined basic concepts. In actual fact no science, not even the most exact, begins with such definitions. The true beginning of scientific activity consists rather in describing phenomena and then in proceeding to group, classify and correlate them. Even at the stage of description it is not possible to avoid applying certain abstract ideas to the material in hand, ideas derived from somewhere or other but certainly not from the new observations alone. Such ideas—which will later become the basic concepts of the science—are still more indispensable as the material is further worked over (Freud, 1915)” (Instincts and their Vicissitudes. 1915).

Sigmund Freud established psychoanalysis, which continued to develop and spread worldwide within and outside psychiatry until the 1970s. At the same time, neuroscience and biological psychiatry have followed their own developmental paths. Psychopharmacological treatments had become widely accepted for mental illness since the 1970s and by the 1980s, psychoanalysis was regarded to be outdated, even unscientific (Wolpert and Fonagy, 2009; Fonagy and Lemma, 2012; Salkovskis and Wolpert, 2012). However with the rethinking of Freudian concepts, neuroscience has recently started to refocus upon psychoanalytic theories in the novel field of neuropsychoanalysis (Fonagy, 2001; Solms and Lechevalier, 2002; Solms and Turnbull, 2002; Panksepp, 2007; Arminjon et al., 2010; Northoff, 2011; Panksepp and Solms, 2012).

**Figure 1:**
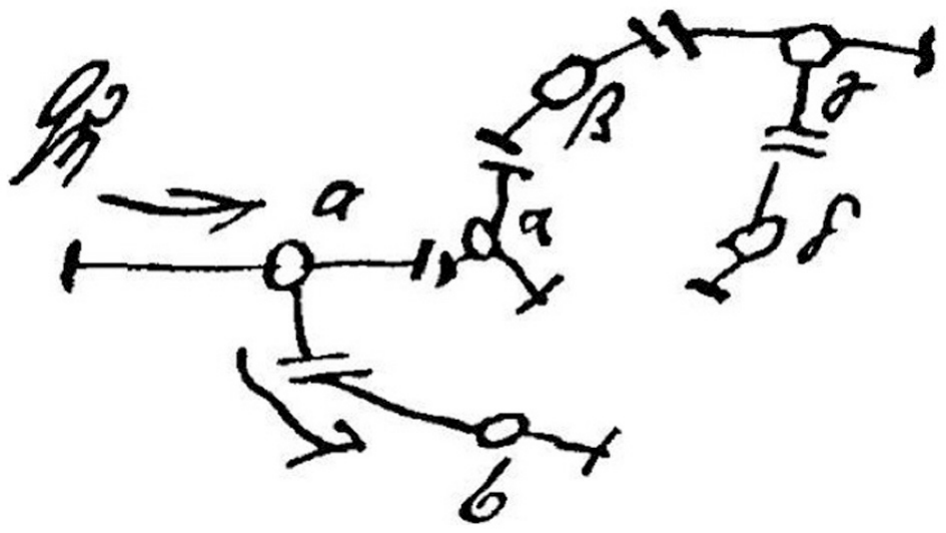
**Freud's sketch of the neuronal mechanism of the mind.** Freud proposed a neuronal mechanism of repression in 1895. This is the famous sketch of his idea. He forecasted the existence of the “synapse” in this sketch. According to his scheme of unpleasant memory, a stimulus (Qη) normally activates unpleasant memory from neuron “a” to neuron “b”; however if other neurons (“α” and “β”) exert a “repressing” influence, such activation is prevented. Based on our microglia theory, the function of “Qη” and/or “α” could be equivalent to a function of microglia as an energy source. Not only “repression” but also other unconscious functions, which were discovered by Freud and later psychoanalysts, may be modulated by microglia.

As a matter of fact, Freud himself began his career as a neurologist, and was a leading neuroscientist in the late 19th century before his establishment of psychoanalysis. His neuronal network idea at that time can actually be found in his private letters to Wilhelm Fliess. Charles Scott Sherrington, a famous British physiologist, discovered gaps between neurons and called them “synapses” in 1887. Two years before the Sherrington's discovery, Freud sketched synapse-like drawings and the existence of a energy source in his private letter (Figure 1) (Freud, 1950 [1895]). This fact highlights Freud's foresight in neuroscience. However, after he had established psychoanalysis, he devoted himself to developing not neuroscientific but psychological theories, and never published his schemes of neurons during his life (Northoff, 2012). Could it be possible that Freud might have dreamed of biological explanations of the mind that would one day replace psychological ones? If he had lived in our modern era, he might have proposed such a hypothesis to modern neuroscientists.

## Is the mind a computer? do computers need energetic drives?

Various hypotheses have been investigated to clarify the relationship between the mind and the brain; however the underlying mechanism remains unresolved. Traditionally, from a neuroscience perspective, the mind has been regarded to consist of neurons and neuronal circuit systems including synapses in the brain in the same way that computers consist of intricate metallic circuitry, and this view has persisted to the present day. In addition, the pathophysiology of mental illness has also been regarded to be within the context of neuronal circuit disturbances via neurotransmitters. On the other hand, a century ago, Freud proposed an energy model of the unconscious mind initially described as “Qη” (Figure 1) (Freud, 1950 [1895]), although this idea has been greatly ignored within the neuroscientific world. However, the computer itself does not work without energy and will only work adequately when the energy systems, such as heating/cooling, operate appropriately. Similar to a computer system model, additional energy systems in the brain may be needed to operate the mind. Originally, Freud and psychoanalytic researchers conceptualized “energy” similar to the thermodynamic conception of energy that all energy tends to ultimate equalization and stabilization and that, therefore, units of higher energy content within a system of lower energy content are unstable and tend to degrade, by importing physics theories from metapsychological perspectives (Freud, 1895, 1933a; Bernfeld and Feitelberg, 1931; Penrose, 1931; Erdelyi, 1985). On the other hand, recent biological studies have suggested that inflammation and oxidative stress, two of the most important energies in the brain, play important roles in the pathophysiology and interventions of various mental illnesses (Ng et al., 2008; Kato et al., 2011a; Maes et al., 2011, 2012). Herein, we propose a novel theory of an unconscious mind structural system in the brain by importing the role of microglia as the energy source and modulator of the brain from a neuropsychoanalytic approach (Solms and Lechevalier, 2002; Solms and Turnbull, 2002; Panksepp, 2007; Northoff, 2011; Panksepp and Solms, 2012).

## What are microglia?

Microglia, which were initially discovered by del Rio Hortega in 1919, are one of the glial cells in the brain. Traditionally, neuroscientists regarded microglial function as simply providing physical support and maintenance for neurons. Thus, in this limited role microglia had been long ignored (Miller, 2005). The last 20 years have elucidated various biological functions of microglia that act as “brain macrophage”; crucial immunological/inflammatory players in the brain by moving around and releasing cytokines and free radicals (Block et al., 2007; Hanisch and Kettenmann, 2007). Thus, microglia have proved to play more important roles in normal brain functions and various brain pathologies such as neurodegenerative diseases and neuropathic pain via inducing inflammation and oxidative stress (Inoue and Tsuda, 2009; Graeber, 2010; Graeber and Streit, 2010; Kettenmann et al., 2011; Ransohoff and Stevens, 2011).

## Psychiatric disorders and microglia

Inflammation, oxidative stress, and immunological abnormality have been highlighted in various psychiatric disorders (Ng et al., 2008; Pasco et al., 2010; Kato et al., 2011a; Maes et al., 2011, 2012; Davison, 2012; Nicholson et al., 2012). The pathophysiology of psychiatric disorders has been dominantly believed to be solely explained by abnormalities of neurotransmitter systems. While, recent brain imaging and histological studies have indicated microglial activation in the brain of people with psychiatric disorders such as schizophrenia, depression, and autism (Radewicz et al., 2000; Steiner et al., 2008; Van Berckel et al., 2008; Doorduin et al., 2009; Morgan et al., 2010; Takano et al., 2010). Psychotropic drugs have long been regarded to have effects solely on neurons and neuronal networks including synapses, while our rodent *in vitro* studies have proved the novel effect of psychotropic drugs directly on microglia by suppressing release of inflammatory cytokines and free radicals (Kato et al., 2007, 2008, 2011a,b; Horikawa et al., 2010). Based on the above-mentioned findings, we have proposed a microglial contribution to psychiatric disorders (Monji et al., 2009; Kato et al., 2011a). Immunological/inflammatory activators such as lipopolysaccharide (LPS) and interferon-γ, which are induced by infections, and various stressful life events, may activate microglia in the brain. Activated microglia release proinflammatory cytokines and free radicals (Block and Hong, 2005). In the brain of patients with psychiatric disorders, these mediators may cause brain pathologies such as neuronal degeneration, white matter abnormalities, and decreased neurogenesis (Uranova et al., 2004, 2007; Jarskog et al., 2005; Lieberman et al., 2005; Girgis et al., 2006; Glantz et al., 2006; Macritchie et al., 2010). Such remodelings of neuron-microglia interactions may thus be important factors in the pathophysiology of psychiatric disorders (Monji et al., 2009, 2011; Kato et al., 2011a).

## Stress, suicide, and microglia

Furthermore, recent animal studies indicate that microglia are activated not only under inflammation but also under physical stress (Frank et al., 2007; Sugama et al., 2007, 2009) and under psychosocial stress such as social isolation (Schiavone et al., 2009), chronic restraint stress (Tynan et al., 2010; Hinwood et al., 2012a,b) and social defeated situations (Wohleb et al., 2011). These data suggest that microglia may contribute not only to physical disturbance but also to emotional disturbance. Human postmortem studies have revealed microglial activation in the brain of suicide victims (Steiner et al., 2006, 2008). Suicide has generally been regarded as a byproduct of emotional disturbance, and furthermore, in the field of psychology and psychoanalysis, suicide has been considered to be the result of maladaptive unconscious drives. Herein, the question arises: Could microglia drive our unconscious drives? Before presenting a bridging theory between microglia and unconscious drives, we introduce the historical concept of these psychoanalytic drives.

## The concept of psychoanalytic unconscious drives

A century ago, Freud proposed the conception of mind structure models consisting of the following three components: *the id* (unconscious/instinctual drives), *the ego* (the exclusive apparatus of the conscious mind), and *the super ego* (which represses *the id* in order to avoid any disruptions of rational thought). In the process of clarifying the unconscious components—*the id* and *the super ego*, Freud additionally developed the economic energy models of the following unconscious drives; first the “*life instinct (Lebenstrieb)*”—the tendency toward survival, propagation, and other creative life-producing drives, and later the “*death drive (Todestrieb)*” described in “*Beyond the Pleasure Principle* (Freud, 1920)” as “*… everything living dies for internal reasons—becomes inorganic once again—then we shall be compelled to say that ‘the aim of all life is death’ and, looking backwards, that ‘inanimate things existed before living ones’*”.

Following Freud's discovery of *the death drive*, it has continued to be one of the key concepts of psychoanalysis, which is often considered to form the basis of various emotions/behaviors—anxiety, fear, aggression and envy, and problematic behaviors including violence and suicide (Freud, 1933b; Klein, 1957). Historically, Freud underpinned *the death drive* from clinical phenomena such as negative therapeutic reactions, repetition-compulsion, anxiety dreams in persons with war neurosis, and masochism. Freud considered that *the life instinct* and *the death drive* fuse together in early life stages, and emphasized that *the death drive* was silently driving individuals toward death and that only through the activity of *the life instinct* was this death-like force projected outwards and appeared as destructive impulses directed against objects in the outside world (Freud, 1924). Freud named the outward-directed death drive “the destructive instinct (drive).” Melanie Klein and Karl Menninger were among the very few psychoanalysts who succeeded and developed the concept of *the death drive*. Klein, the Vienna-born British female psychoanalyst, who further developed Freud's concept of *the death drive* and was the basis of the Kleinian school in her later life, regarded the super ego in early life stages as the clinical expression of *the death drive* (Klein, 1932). Based on her theory, humans genetically and potentially have both *the life instinct* (desires for affection and/or objects) and *the death drive* (destructiveness and aggression), and these drives are expressed as internal/external object relations (good object/bad object) (Klein, 1957). Klein and Hanna Segal, a prominent Kleinian psychoanalyst, linked *the death drive* to envy (Segal, 1952, 1993). Segal also linked it to aesthetics by describing that “*Re-stated in terms of instincts, ugliness—destruction—is the expression of the death instinct; beauty—the desire to unite into rhythms and wholes, is that of the life instinct. The achievement of the artist is in giving the fullest expression to the conflict and the union between those two* (Segal, 1952).” Herbert Rosenfeld regarded the death drive in line with the concept of the *pathological organization* (*narcissistic organization*) in which good objects are abolished and destroyed internally in the self (Rosenfeld, 1971). As stated above, Kleinian theory has been continuously developed based on two opposing internal objects; the good object and the bad object. On the other hand, independent group psychoanalysts have developed their own theories. Ronald Fairbairn avoided the good/bad dichotomy, and established a unique object-relation theory with two essential objects; the exciting object and the rejecting object (Fairbairn, 1952). He assumed that the two internal objects were the roots of human behaviors and emotional life. Donald Winnicott emphasized the importance of external objects (environmental factors) in addition to internal objects (Winnicott, 1953, 1960).

Researchers such as Heinz Hartmann, Otto Kernberg, and Jaak Panksepp have fundamentally discussed the concept of instincts and drives in psychoanalysis in connection with biology and affective neuroscience. Hartmann, one of the founders of ego psychology, developed the theory of aggression based on *the death drive* (Hartmann, 1939). In addition, Panksepp, who coined the term “affective neuroscience,” has been proposing a provocative theory linking drives and emotions. Based on his neurobiological and neuropsychoanalytic background, he and his colleagues have recently developed the theory of the SEEKING system (Wright and Panksepp, 2012). The SEEKING system is described as a “primary process” that promotes psychomotor eagerness to obtain pleasure generating resources and eliminate calamities, providing euphoric anticipatory excitement, and linking with other drives, such as those apart of the rewarding affective systems of LUST, CARING, and PLAY, and at times the aversive affective systems of FEAR and RAGE (Wright and Panksepp, 2012). Interestingly, in the commentary of the article of Wright and Panksepp, and Kernberg suggested “the concept of ‘death drive’ be retained for the pathological predominance in some clinical conditions of negative internalized object relations that may lead to an overwhelming dominance of self-destructive motivation (Kernberg, 2012).”

In psychoanalysis, the relationship between Es (id), libido and drive (instinct) has been ambiguously classified. While valuing Freud's original concept of the two essential drives and the following psychoanalytic theories, we believe that these concepts should be modified with accordance to recent theoretical/biological developments as discussed above. In the present day, the majority of psychoanalysts and scholars such as ethologists and experimental psychologists are skeptical regarding the validity of *the death drive* as a relevant concept (Dufresne, 2000), but many researchers continue to accept the concept of the (aggressive) destructive drive (Rosenfeld, 1971; Feldman, 2000; Britton, 2003; Kernberg, 2012). In this article, we use the term of *the death drive* basically as the destructive drive (instinct), which induces negative emotions and outward destructive behaviors. In the following part, we propose a novel integrating theory of unconscious drives in order to fit both psychoanalytical and biological models.

## Bridging theory between microglia and unconscious drives—do microglia drive human mental activities as the origin of unconscious drives?

To our knowledge, the internal reasons of the death drive have never been clarified from a molecular neuroscientific perspective. We herein propose a novel challenge to dig up the underlying mechanism of the drives with the modern understandings of microglia and their immunological roles in the brain. Obviously, Freud would not have known of such cells, however surprisingly, he implied a linkage between immunity and suicide in the following sentence:

“… It is noteworthy that the obsessional neurotic, in contrast to the melancholic, never in fact takes the step of self-destruction; it is as though he were *immune* against the danger of *suicide*, and he is far better protected from it than the hysteric (Freud, 1920).”

In the present day, the role of microglia has been understood with a greater clarity than in Freud's era. Synaptic reactions have for a long time been regarded to play an essential role in human mental activities, while only neurons have been highlighted. Now, rodent microglia have proved to contribute to brain development such as synaptic pruning (Paolicelli et al., 2011), which suggest that microglia may play an important role in the process of brain development. Other animal studies have shown that microglia monitor synaptic reactions via direct-touching even in the normal brain (Wake et al., 2009; Graeber, 2010; Ransohoff and Stevens, 2011). Interestingly, some synapses in the ischemic areas disappear after a prolonged microglial contact (Wake et al., 2009), which may suggest that severe mental stress induces synaptic changes via microglial responses. Recent rodent studies have reported that severe stresses including psychosocial stress activate microglia (Frank et al., 2007; Schiavone et al., 2009; Sugama et al., 2009; Tynan et al., 2010; Wohleb et al., 2011; Hinwood et al., 2012a,b). In addition, human studies suggest that microglial activation is observed in the brain of psychiatric patients and suicide victims (Steiner et al., 2006, 2008; Van Berckel et al., 2008; Doorduin et al., 2009; Takano et al., 2010). Under these microglia-activated states, unconscious drives could be highly activated from a psychoanalytic perspective.

**Figure 2:**
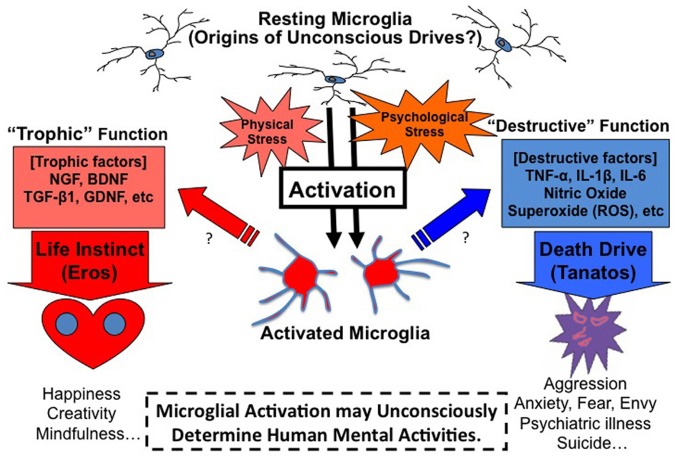
**Microglia theory of unconscious drives.** Microglia may act as the origin of unconscious drives in the mind. Microglia play the role of a double-edged sword in the brain. Microglia release not only maladaptive factors such as TNF-α but also protective factors such as BDNF, which means that microglia are alternately both bad and good actors in the brain. The direction and strength of microglial activation may be the origin of Freud's two essential drives of death (tanatos) and life (eros). “Destructive” function of microglia may play an essential role in *the death drive*. Maladaptive microglial activation caused by a certain psychological/physical stress may induce aggressive, anxious, fearful, and envious states: these states may furthermore lead to psychiatric disorders and suicidal thought/behaviors. On the other hand, “Trophic” function of microglia may play an important role in *the life instinct*. Appropriate stress may activate microglia at good enough levels, which induce protective factors and result in happy, creative and mindful states.

In sum, a novel hypothetical theory arises: “When microglia is maladaptively activated in the brain, microglia may act as the origin of unconscious drives such as *the death drive* in the unconscious mind, and induce emotional reactions such as anxiety, fear, aggression, envy, and suicidal thought/behaviors (Figure 2).”

## Transference, psychological immune memory/reaction, and microglia

One of the essential lessons of psychoanalysis represented by the Oedipus complex is that psychological experiences during childhood between people closely related (i.e., mother, father and siblings) last until adulthood, (Freud, 1900, 1905). Unconscious reactions, which are memorized during childhood, are reflected onto immediate persons in various settings such as home, school, and work. These unconscious reactions occurring toward psychoanalysts are called transference; e.g., a client felt enraged toward his psychoanalyst, as he would have experienced toward his father in childhood. Dealing with transference is a major therapeutic approach of psychoanalysis. Psychoanalysts would interpret that his unconscious aggressive drive produced by the father–child relationship is reproduced during the here and now psychoanalyst-client relationship. Owing to such an approach, the client may recognize his own unconsciously derived aggression and he may be released from it.

Transference and its underlying mechanisms can be explained within the paradigm of microglial priming. Bilbo and Schwarz suggest that microglial activation due to infections during early developmental periods last, and these pre-activated microglia will be re-activated rapidly compared to normal state microglia as microglial immune memory (Bilbo and Schwarz, 2009). Interestingly, Bilbo and her colleagues recently reported that early life stress in the rat influence formation of memories in later life by microglial immune memory (Williamson et al., 2011).

**Figure 3:**
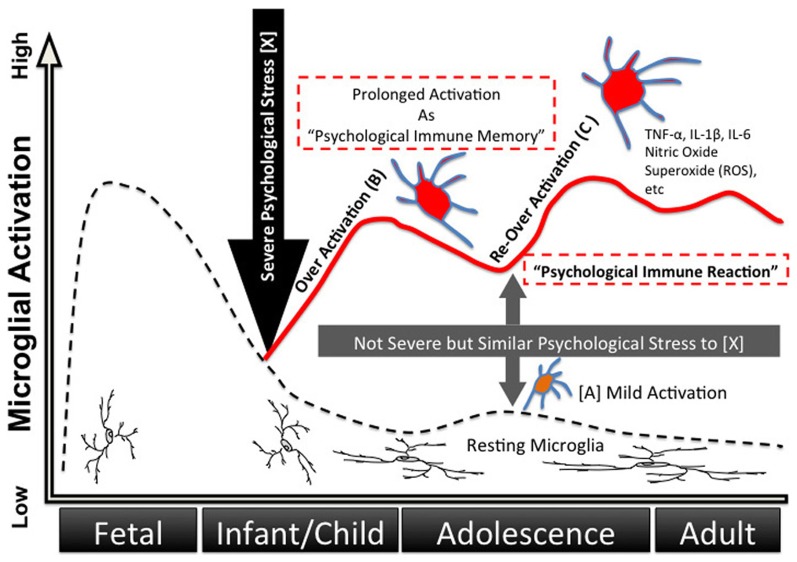
**Developing process of “psychological immune memory/reaction” via microglia.** Microglia have proven to have direct connections with neuronal synapses, and external stress activates microglia via neuronal stimulus. Not only physical stress but also psychological stress activates microglia in animal models. Herein we propose a possible process of microglia activation as “psychological immune memory” during human life. **(A)** Normal daily psychological stress (not extreme stress) moderately and temporarily activates microglia via neuronal stimulus **(A). (B)** Severe psychological stress (e.g., trauma) over-activates microglia via nervous excitement **(B)**, which induces a storm of inflammatory cytokines and free radicals in the brain, thus resulting in abnormal behaviors and strong emotional reactions. In addition, this storm results in damaging neuron-glial networks and the subsequent rebuilding of novel neuro-glial networks. This network change means that previous psychological reactions dramatically change into novel stimulus-output patterns. Moreover, once microglia have been activated strongly, these microglia remain in pre-activated states for years, which we can dub “**psychological immune memory.**” **(C)** Pre-activated microglia **(B)** are excessively responsive to even slight stimuli when the stimuli are similar to the previous traumatic stress, and are again over-activated **(C)**. Similar but slight stress, which was previously only a good enough modulator of microglia, results in a strong storm of the brain/abnormal behavior/strong emotional reaction as that of the previous traumatic reaction. We have dubbed this reaction “**psychological immune response,**” and it can explain many psychological and psychopathological mechanisms such as transference and repeated behavioral/emotional reactions typically seen in PTSD.

**Figure 4:**
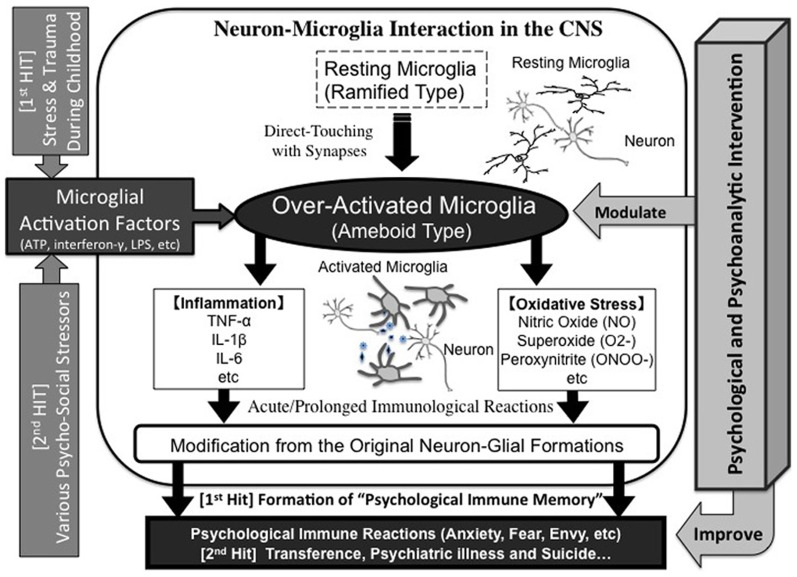
**Neuron-microglia interaction during “psychological immune memory/reaction.”** Strong psychosocial stress such as trauma during childhood may over-activate microglia, which induce a variety of inflammatory/oxidative-stress factors and result in damaging the original neuron-microglial formations. Finally, novel neuron-microglial networks will be formulated. This reaction is memorized as “psychological immune memory”. When similar psychosocial stress, even at weaker levels, occurs in later life, the primed-microglia may be over-activated. This reaction will also induce various maladaptive psychological reactions, which may result in transference reactions during interpersonal relationships, psychiatric disorders and also suicidal behaviors. On the other hand, psychological and psychoanalytic interventions may improve these states by suppressing microglial maladaptive activation.

Various stressors, not only infection but also psychosocial stress, may be memorized inside the microglia during childhood as the origin of unconscious drives, which we have dubbed “psychological immune memory.” In later life, various similar stressors re-activate the microglia and lead to transference-like situations; emotional reactions during childhood (i.e., traumatic events) are reproduced afterwards as “psychological immune reactions” (Figures 3 and 4). The underlying mechanism of Post-Traumatic Stress Disorder (PTSD) could also be explained by this process. Interestingly, Klein proposed the “memories in feelings” in her representative book “Envy and Gratitude (Klein, 1957).” The word “memories in feelings” means that strong primitive feelings themselves during childhood are memorized psychologically, and these feelings are reenacted in later life as transference. Klein described such feelings as follows:

“All this is felt by the infant in much more primitive ways than language can express. When these pre-verbal emotions and phantasies are revived in the transference situation, they appear as ‘memories in feelings’, as I would call them, and are reconstructed and put into words with the help of the analyst. In the same way, words have to be used when we are reconstructing and describing other phenomena belonging to the early stages of development. In fact we cannot translate the language of the unconscious into consciousness without lending it words from our conscious realm (Klein, 1957).”

This Kleinian mechanism may also be explained by our microglia theory of the psychological immune memory/reaction. Recent epidemiological studies have revealed that maladaptive parent-child relationships and childhood trauma are the crucial risk factors for psychiatric disorders in later life (Alvarez et al., 2011; Bebbington et al., 2011; Hovens et al., 2012; Morgan et al., 2012). In addition, a recent report of a human twin study suggests that childhood trauma induces inflammatory reactions (Rooks et al., 2012). Such evidence supports our proposed theory that microglial immune memory may develop psychiatric disorders in later life.

The origin of unconscious processes in the brain especially in psychiatric condition has not been well understood. Our theory may reflect a heightened attempt by microglia to achieve homeostasis in the brain when it is under physical or psychosocial stress. In the process of understanding emotional systems in the brain, neuronal centered explanations have been dominant including the importance of schemata, higher-order conditioning, implicit memory, and experience-dependent shaping of neurotransmitter systems (Solms and Turnbull, 2002; Panksepp, 2004; Welzer and Markowitsch, 2005; Wright and Panksepp, 2012). At present, the connection between the immunological role of microglia and our proposed “psychological immune memory” has not been well clarified. However, a series of studies by Bilbo and her colleagues (Bilbo and Schwarz, 2009; Williamson et al., 2011) and other recent thought-provoking animal studies have suggested interesting physiological outcomes regarding microglial contribution to psychological immune memory and emotional responses. As shown the above, rodent studies have reported that severe stresses including psychosocial stress activate microglia (Frank et al., 2007; Schiavone et al., 2009; Sugama et al., 2009; Tynan et al., 2010; Wohleb et al., 2011; Hinwood et al., 2012a,b). Acute stress is demonstrated to induce morphological microglial activation in several brain regions including the midbrain periaqueductal gray (PAG), an area that plays crucial roles in behavioral and emotional responses to uncontrollable stress, threat, anxiety, and pain. Sugama et al. determined whether neuronal activation may be involved in the stress-induced microglial activation by measuring the correlation between neuronal activity measured as c-Fos expression and morphological microglial activation in the PAG (Sugama et al., 2009). Acute stress was succeeded by morphological activation of microglia and increased c-Fos expression in the PAG, and their analysis demonstrated that microglial activation occurred adjacent to responsive neurons. By contrast, LPS treatment induced microglial activation even in the absence of neuronal responses in the PAG as well as in the rest of the midbrain. Their findings suggest that the mechanism of microglial activation during stress may differ from those of infection or inflammation. Based on their results, Sugama et al. suggested that stress-induced c-Fos protein from activated neuronal cells may play some roles to trigger microglial activation. Recently, Hinwood et al. investigated a series of rodent studies how psychological stress affects microglia (Hinwood et al., 2012a,b). They found that chronic psychological stress increases the internal complexity of microglia, and that chronic stress markedly increases the expression of beta1-integrin (CD29), a protein previously implicated in microglial ramification (Hinwood et al., 2012b). These findings suggest that beta1-integrin may be one possible modulator between psychological stress, neuronal network activity and microglial ramification (Hinwood et al., 2012b). Above-mentioned animal studies indicate that unconscious drives may involve both activated neurons and/or activated microglia, while it is very difficult to differentiate between clusters of neuronal activation and microglial activation in the process of unconscious brain processes and emotional motivations because of the difficulty of establishing experimental models. Furthermore, to our knowledge, it is also difficult under current scientific conditions to clarify whether microglia are the underlying precipitator of unconscious thought processes and motivations. To our knowledge, the exact process of how microglial and/or neuronal activation affect emotional experience and behavior has not been well understood. Interestingly, a recent animal study has suggested that microglial activation has a positive link to anxiety-like behaviors, and suppressing microglial activation by minocycline results in ameliorating the anxiety-like behaviors (Neigh et al., 2009). This report suggests that microglial activation may, at least to some extent, contribute to the occurrence of anxiety. As introduced the above, microglia are recently known to have continuous direct contact with synapses (Wake et al., 2009). In addition, microglia are known to have various neurotransmitter receptors, and neurotransmitters are reported to affect not only the neuronal system but also microglia (Pocock and Kettenmann, 2007; Kato et al., 2013). Therefore, in our opinion, microglial activation may induce a disturbance of neuron-microglia communication at least to some extent, and neuronal systems, which organize emotional and psychological experience and behavior, may be over-activated. Further studies should be conducted to clarify how microglial activation affects neuronal system, emotional and psychological experience and behavior.

Microglial psychoimmunological memory is a novel concept which we have just recently proposed. To our knowledge, no study has been conducted in this aspect. Traumatic memories may be located within neural networks without having to recur to microglia, or microglia may contribute much to such memories. Further studies are needed to clarify the relationship between early trauma, emotional behavior and microglial immune reactions.

## Social interaction and microglia in healthy human

Until recently no experiment had been conducted focusing on human social and psychological factors in relation to microglia, and there is no known drug with the specific effect of modulating human microglia. Therefore, using minocycline, a tetracycline antibiotic and the most famous microglial inhibitor in rodent models, is one of the best alternative approaches to clarify microglial functions in human social/mental activities. A recent rat study has shown that minocycline suppresses microglial activities not only in stress-induced activation states but also in resting states (Hinwood et al., 2012b). In order to examine how microglia influences social and mental activities, we recently examined how minocycline works in human social decision-making by trust game (Watabe et al., 2012); healthy adult males made a monetary decision about whether or not to trust an anonymous partner after a 4-day oral administration of minocycline. The minocycline group showed a positive correlation between their monetary score in the trust game and their evaluation scores of others' trustworthiness in a questionnaire, but surprisingly the placebo group did not. Thus, minocycline sharpened participants' sense of trust that led them to be more decisive in the game. This first trial has suggested that microglial activation may cause “unconscious noises” against appropriate social decision-making, and inhibiting microglial activity may reduce such noise (Watabe et al., 2012). In a subsequent trial with larger samples, we additionally measured the effects of anxiety and personality (Kato et al., 2012). The monetary score in the trust game was significantly lower in the minocycline group. Interestingly, participants' ways of decision-making were significantly shifted; cooperativeness, one component of personality, proved to be the main modulating factor of decision-making in the placebo group, on the other hand, the minocycline group was mainly modulated by state anxiety and trustworthiness. Our results of the second trial suggest that minocycline led to more situation-oriented decision-making, possibly by suppressing the effects of personality traits, and furthermore that personality and social behaviors might be modulated by microglia. Early life events may activate human microglia, establish a certain neuro-synaptic connection, and this formation may determine personality and personality-oriented social behaviors in later life (Kato et al., 2012).

The above-mentioned findings shed new light on the dark side of microglial social/mental functions in humans, especially highlighting the role of microglia for the unconscious. In the same way that Freud proposed that our behaviors must be controlled by the unconscious world, microglia may unconsciously control our behaviors. Human neuroscience focusing not only on computer-like neuronal networks but also on “noisy” microglia would be a novel key for investigating “noisy” human social/mental activities that are unlike “noiseless” computers. To explore these mechanisms, further translational research is needed.

## Microglial double-edged sword and ambivalence

Microglia play an interesting role as a double-edged sword in the brain (Henkel et al., 2009; Graeber and Streit, 2010). Microglia release not only maladaptive factors such as Tumor Necrosis Factor (TNF)-α but also protective factors such as Brain-Derived Neurotrophic Factor (BDNF), which means that microglia are alternately both bad and good actors in the brain. “Destructive” function of microglia may play a vital role in *the death drive*. On the other hand, “trophic” function of microglia may play an equally essential role in *the life instinct*. It remains controversial as to whether the origin of the two drives is the same from the psychoanalytic perspective. Based on our microglial theory, the origin and the determinant factor may be the composition and the direction of the microglia. Microglia are known to express different *faces* during developmental, adolescent and adult stages. The balance-shift of the trophic/destructive expression of microglia may explain the underlying origin of the two drives in the mind. The existence of two directional microglia in the same region may induce an ambivalence, which means a dilemma between the two directional emotions such as “love and hate.” The direction of microglial activation may determine our behaviors toward life or death (Figure 2).

Our terms “trophic” and “destructive” microglia should not be taken in a strictly literal sense. Our proposed theory may be too oversimplified in implying that the function of microglia easily divides into (A) “trophic” function of microglia = preserving = the life instinct, and (B) “destructive” function of microglia = destroying = the death instinct. This dichotomy is not always true in real situations. Some microglia might destroy for synaptic pruning, which in the long run is a trophic result for the brain, to preserve energy for more frequently functioning neuron populations, and to reconstruct more appropriate neuronal networks. Furthermore, we could apply this proposed neuroscientific process into human psychological development as follows: It is somewhat essential to have painful/stressful experiences in developing periods, during which microglia may activated, and neuronal networks may be reformed, and finally rebuilt a more prosocial personality and/or resilient self in later life. However, for some, this process might not work through, and result in pathological/psychiatric conditions. It is not known how differentiate destructive processes that are useful, from destructive processes that are associated with pathology, while we prospect that these different outcomes might be determined by factors such as genetic vulnerability, extremely painful/stressful events, dysfunction of neurons/microglia, and environmental factors before/after these events. For example, some volume of microglia-releasing mediators such as pro-inflammatory cytokines and/or free radicals may be essential for our mental development; however microglia in some individuals may easily release too much of such mediators even after weak stressful events. Those individuals may easily be prone to psychiatric conditions. At least to some extent, recent neuropsychoanalytic theories such as the Panksepp's SEEKING system (Wright and Panksepp, 2012) and Kernberg's “death drive” theory (Kernberg, 2012) may be complemented by our proposed microglial theory. Digging up these interactions provide for further translational research opportunities to bridge the huge gap between the brain and the mind. Aging is known to be one of the key switching factors of microglial characteristics. Generally speaking, aging tends to activate microglia maladaptively (Dilger and Johnson, 2008; Jang and Johnson, 2010; Norden and Godbout, 2012), which may provide a clue to clarify these underlying mechanisms.

## Possible microglial contribution to the conscious and the unconscious world

It is of great importance to understand the present situation of affective neuroscience and neuropsychoanalysis including the biological understanding of the unconscious/conscious. To our knowledge, all previous research has been focused solely on neuronal systems including synapses to understand the emotional reactions and the unconscious in the brain. It is a novel challenge to consider the role of microglia in emotional reactions and the unconscious. Neuronal systems and neurotransmitters have been regarded to have important roles in “unconsciously” modulating emotions and motivational behaviors (Solms and Turnbull, 2002). In addition, microglia may be one possible source of “unconsciously” generated negative emotions that do not directly rely on perceptual input but are generated biologically. Herein we hypothesize a possible role for microglia in emotional reactions. The following three processes might be occurring at least in some biological pathways of the unconscious/conscious; (Process I) microglia may be activated by neurotransmitter modulations connected with emotional reactions based on perceptual inputs, (Process II) microglial activation may modulate synaptic reactions via neurotransmitters resulting in emotional reactions, and (Process III) a mixed process of I and II may occur especially during continuous high emotional responses, in which primary emotional reactions may activate neuronal systems via synapses and neurotransmitter modulations, resulting in microglial activation, and finally mutual activation may occur via neurotransmitters and microglial mediators such as free radicals and/or cytokines. We hypothesize that process III may be one of the possible causes for emotional disturbance, symptoms of various psychiatric disorders and also suicide.

In addition, we now present a possible mechanism of the conscious and the unconscious in the brain. The system of the relationship between the conscious and the unconscious has long been considered within the context of neuronal systems. Microglia are now known to be very unique dynamic cells in the brain, which can move around and are usually independent from neuronal systems, and sometimes have direct contact with synapses. These roles seem to be similar to Freud's perceptual theory called “the system Pcpt.-Cs., or the system W-Bw, which was named after the German words Wahrnehmung (= perception; Pcpt.) and Bewußtsein (= consciousness; Cs.)” (Freud, 1920). We suppose that microglial activation itself does not directly equate to emotional reactions, but we suggest that microglial activation may be one of the crucial priming factors of the unconscious for emotional reactions by affecting neuronal systems. It is easily understood that external inputs trigger emotional reactions, while the mechanism of emotional reactions without external input such as nightmares has not been fully comprehended. Our theory may shed new light on the understanding of internally caused (or the unconscious-derived) emotional reactions. Interestingly, microglial contribution has recently been suggested in the occurrence of delirium, which induces disturbance of the conscious by internal causes such as systemic infections (Van Gool et al., 2010). Our theory might give us the chance to re-translate Freudian theory of the system between the conscious and the unconscious. Further studies should be highlighted in this aspect.

## Conclusion

### Future perspectives

In this paper, we showed the possibility that microglial activation in the brain activates unconscious drives in the mind. We also presented the brain/mind structural system of ambivalence, transference, psychological trauma and even the Oedipus complex by importing the microglia theory of “psychological immune memory/reaction.” In addition, we introduced a recent human study focusing on the microglial role of social decision-making. Finally, we showed a possibility that direction and context of microglial activation may be a key factors in our mental activities including unconscious world.

Previously, Eric Kandel explored the neuron-synaptic world based on his psychoanalytic background as a novel work of the 20th century (Kandel, 1979, 1999, 2005). In a similar mode to Kandel's exploration, the novel scientific field, now highlighted as “neuropsychoanalysis” (Fonagy, 2001; Solms and Lechevalier, 2002; Solms and Turnbull, 2002; Panksepp, 2007; Northoff, 2011; Panksepp and Solms, 2012), has endeavored to clarify the underlying mechanism of the unconscious and psychoanalytic theories from a neuroscientific perspective. In the 21st century, new challenges focusing on microglia should be explored in the new world of the mind/brain beyond Kandel's neuron-synaptic doctrine. We believe that our proposed theory sheds new light on solving deeper mechanisms of “unconscious drives” from both psychoanalytic and neuroscientific perspectives. Microglia may have the potential to bridge the huge gap between neuroscience, biological psychiatry, psychology, and psychoanalysis. Further communication between neuroscientists, psychiatry, psychologists, and psychoanalysts is required. To investigate the microglia theory, further translational research from *in vitro*/*in vivo* animal studies to human studies is needed based on the neuropsychoanalytic approach. Finally, we highlight some research questions of particular importance to be clarified:

- What is the key interaction between microglial activation (biological world) and the unconscious (psychological world)?
- What kind of afferent networks (afferent stimulus, input, impulse, etc.) and molecules such as neurotransmitters activate microglia under psychosocial stress?
- How do activated microglia act on efferent neuronal pathways, and how do microglia finally impact on the unconscious, emotions and behaviors? In relation to cognition, various studies suggest the positive link between microglial activation and dementia which is one of the most typical phenotypes of cognitive dysfunction, while the underlying mechanism between dementia's cognitive dysfunctions and microglial activation are less well understood. Can microglia modulate various cognitive functions under not only pathological states but also normal states? It is also unclear how microglia activation influences neurotransmitters and/or neural systems involved in emotional and experience and behavior and how microglial activation back-project to the mental and behavioral realm, while the following evidence may give a cue for future investigations. Not only neurons but also microglia have a variety of neurotransmitter receptors including dopamine and noradrenaline receptors (Pocock and Kettenmann, 2007; Kato et al., 2013), which are closely related to our mental activities and the pathophysiology of neuropsychiatric disorders. Sugama et al. showed that acute stress activates microglia in the PAG (Sugama et al., 2009). In addition, Neigh et al. suggested that microglial activation induce anxiety-like behaviors in mice (Neigh et al., 2009). These reports suggest that microglial activation may contribute to various emotional reactions.
- Microglia are thought to be a heterogeneous group. Therefore, we should investigate the actions of microglia in each group. Regional specificity might exist, and it may link to previously known understandings in psychiatric brain imaging studies.
- Microglial modification may create a novel strategy for intervention in psychiatric disorders. Clinical trials focusing on microglia should be conducted.
- Microglia have mutual communications not only between neurons but also astrocytes and oligodendrocytes. Therefore, mutual interaction of neuron-glia should be clarified to understand the deeper mechanisms of unconscious and neuropsychoanalytic theory in the brain.

## Final remarks

Before developing psychoanalysis in the late 19th century, Freud sketched the neuronal mechanism of the mind (Figure 1), and Freud might have possibly dreamed that biological explanations of the unconscious mind would one day replace psychological ones (Freud, 1950 [1895]). Microglia may be a key player to realize Freud's long-unresolved dream.

### Conflict of interest statement

The authors declare that the research was conducted in the absence of any commercial or financial relationships that could be construed as a potential conflict of interest.
